# Transcriptomic Analysis Identifies Oxidative Stress-Related Hub Genes and Key Pathways in Sperm Maturation

**DOI:** 10.3390/antiox14080936

**Published:** 2025-07-30

**Authors:** Ali Shakeri Abroudi, Hossein Azizi, Vyan A. Qadir, Melika Djamali, Marwa Fadhil Alsaffar, Thomas Skutella

**Affiliations:** 1 Department of Cellular and Molecular Biology, Faculty of Advanced Science and Technology, Tehran Medical Sciences, Islamic Azad University, Tehran 14778-93855, Iran; alishakeriabroudi@gmail.com; 2 Department of Stem Cells and Cancer, College of Biotechnology, Amol University of Special Modern Technologies, Amol 46158-63111, Iran; 3 Department of Basic Science, Faculty of General Medicine, Koya University, Koya KOY45, Iraq; vyan.asad@koyauniversity.org; 4 Department of Biology, Faculty of Science, Tehran University, Tehran 44001, Iran; mdjamali@ut.ac.ir; 5 Medical Laboratories Techniques Department, College of Health & Medical Techniques, AL-Mustaqbal University, Hillah 51001, Iraq; marwaalsaffar@uomus.edu.iq; 6 Institute for Anatomy and Cell Biology, Medical Faculty, University of Heidelberg, Im Neuenheimer Feld 307, 69120 Heidelberg, Germany; thomas.skutella@uni-heidelberg.de

**Keywords:** oxidative stress, sperm, immunocytochemistry, spermatogonial stem cells, microarray

## Abstract

Background: Oxidative stress is a critical factor contributing to male infertility, impairing spermatogonial stem cells (SSCs) and disrupting normal spermatogenesis. This study aimed to isolate and characterize human SSCs and to investigate oxidative stress-related gene expression, protein interaction networks, and developmental trajectories involved in SSC function. Methods: SSCs were enriched from human orchiectomy samples using CD49f-based magnetic-activated cell sorting (MACS) and laminin-binding matrix selection. Enriched cultures were assessed through morphological criteria and immunocytochemistry using VASA and SSEA4. Transcriptomic profiling was performed using microarray and single-cell RNA sequencing (scRNA-seq) to identify oxidative stress-related genes. Bioinformatic analyses included STRING-based protein–protein interaction (PPI) networks, FunRich enrichment, weighted gene co-expression network analysis (WGCNA), and predictive modeling using machine learning algorithms. Results: The enriched SSC populations displayed characteristic morphology, positive germline marker expression, and minimal fibroblast contamination. Microarray analysis revealed six significantly upregulated oxidative stress-related genes in SSCs—including CYB5R3 and NDUFA10—and three downregulated genes, such as TXN and SQLE, compared to fibroblasts. PPI and functional enrichment analyses highlighted tightly clustered gene networks involved in mitochondrial function, redox balance, and spermatogenesis. scRNA-seq data further confirmed stage-specific expression of antioxidant genes during spermatogenic differentiation, particularly in late germ cell stages. Among the machine learning models tested, logistic regression demonstrated the highest predictive accuracy for antioxidant gene expression, with an area under the curve (AUC) of 0.741. Protein oxidation was implicated as a major mechanism of oxidative damage, affecting sperm motility, metabolism, and acrosome integrity. Conclusion: This study identifies key oxidative stress-related genes and pathways in human SSCs that may regulate spermatogenesis and impact sperm function. These findings offer potential targets for future functional validation and therapeutic interventions, including antioxidant-based strategies to improve male fertility outcomes.

## 1. Introduction

Male factors are either the main or a contributing factor in almost half of all infertility cases; this makes infertility a significant global health concern impacting an estimated 10% of all couples [1]. Azoospermia, defined as the complete absence of spermatozoa in the ejaculate, is a major cause of male infertility and may result from defects in spermatogenesis or obstruction of the reproductive tract. Azoospermia is the most prevalent genetic cause contributing to male infertility, which is a multifactorial clinical illness with complicated genetic components [2]. All of spermatogenesis and a man’s ability to conceive begin with spermatogonial stem cells, or SSCs [3]. Hence, stem cell transplantation is an exciting new development in the treatment of male infertility, and SSCs are seen as a potential substitute for regenerating injured or defective spermatogenesis [4]. However, SSCs are limited in supply, and there is a lack of resources for their long-term culture and growth. Several recent studies in humans and animals have shown that embryonic stem cells (ESCs) may develop into putative primordial germ cells (PGCs) and that these PGCs can then transition into stellate stem cells (SSCs) [5,6]. The complexity and lack of clarity around the induction parameters in these findings or their poor induction efficiency highlight the ongoing difficulty of recreating SSC formation in vitro.

A transcriptional shift occurs across the genome in tandem with dynamic chromatin rearrangement. By identifying particular chromatin states and their associated TFs, chromatin accessibility profiling has emerged as a powerful method for investigating genetic and epigenetic regulation, shedding light on the temporal and spatial dimensions of the pangenomic regulatory landscape of cells and tissues [7,8]. Changes in chromatin accessibility may be understood in relation to these ever-changing regulatory networks, and chromatin accessibility profiling is anticipated to be an effective method for identifying regulatory DNA regions that GRNs [9,10]. Two technologies that map the landscape of chromatin accessibility are the assay for transposase-accessible chromatin with high-throughput sequencing (ATAC-seq) and DNase-seq. Not only does this technique distinguish between various cell types, but it also identifies the chromatin accessibility of relevant genes and potential TF-binding sites, as well as regulatory areas that are distinct to each cell type. As a possible regulator of transcription factors, chromatin accessibility is highly connected to differential gene expression. In order to characterize the change in chromatin accessibility during spermatogenesis, ATAC-seq and ChIP-seq were recently integrated. Sakashita et al. found potential regulatory elements for gene expression unique to spermatogenesis and demonstrated genome-wide, dynamic remodeling of open chromatin during spermatogenesis [9]. Additionally, they discovered that sex chromosomes and autosomes have different chromatin environments throughout spermatogenesis [11]. This provides more evidence that bivalent domain creation and poised chromatin are the driving forces behind the epigenetic modifications that occur across the genome in the latter stages of spermatogenesis. Despite growing evidence that oxidative stress (OS) plays a critical role in testicular function and male fertility, the specific molecular mechanisms by which OS impacts human SSCs remain poorly understood. Most existing studies focus on animal models or later stages of spermatogenesis, leaving a gap in knowledge about the early effects of oxidative damage on SSC maintenance, self-renewal, and differentiation. Moreover, integrative analyses combining gene expression profiles, protein interactions, and signaling pathways in human SSCs under oxidative stress are limited. Addressing these gaps is essential for developing targeted interventions to preserve SSC function and male reproductive health.

Using TF-mediated GRNs constructed during SSC formation, our work aimed to test for variables that trigger ESCs to differentiate into SSCs in vitro. We began by mining chromatin property and gene expression datasets (ATAC-seq, DNase-seq, microarray data) for SSC-specific transcription factors and hub SSC-specific genes [12,13]. Following this, we identified human and mouse SSC-specific TFs that overlap. Then, by comparing gene expression among ESCs, PGCs, and SSCs, important TFs unique to SSCs were identified. Last but not least, we used ChIP-seq data to build essential SSC-specific TF-mediated GRNs and compared the gene expression levels of important SSC-specific TFs in normal and impaired spermatogenesis testis samples. For the purpose of improving the induction efficiency of SSC differentiation from ESCs in vitro, our work offers prospective induction variables.

## 2. Materials and Methods

### 2.1. Ethical Considerations and Study Design

Testicular samples were obtained from three adult males (age range 23–67 years) between October 2016 and September 2017. The experimental design was based on our previously established protocols for testicular tissue research. Ethical approval was granted by the Amol University of Special Modern Technologies (approval code: Ir.ausmt.rec.1403.06) and the Committee of the Medical Faculty of the University of Heidelberg (reference number S-376/2023). All participants provided written informed consent after receiving comprehensive information about the study [8,14,15,16,17].

This investigation compared gene expression patterns between short-term (<2 weeks post matrix selection) SSC cultures and long-term (>2 months, up to 6 months) human adult germline stem cell (haGSC) cultures. Microarray analysis was employed to compare these patterns with human embryonic stem cells (hESCs) and fibroblasts. This pilot study included three individuals due to ethical and logistical constraints related to acquiring fresh testicular tissue. Despite the small cohort, we ensured technical replicates and colony-level sampling to capture intra-donor variability.

### 2.2. Participant Selection Criteria

Participants included healthy adult males of reproductive age with confirmed fertility and no reported fertility issues, based on clinical history and recent conception reports.

Exclusion criteria encompassed history of cancer, mumps, cryptorchidism, infectious or systemic conditions affecting SSCs, medications impacting the reproductive system (including chemotherapy or hormonal treatments), and lifestyle factors known to affect sperm quality (smoking, alcohol abuse, drug use).

### 2.3. Human Fibroblast Cultivation

Primary human fibroblasts were isolated from scrotal dermis and cultured in DMEM high glucose supplemented with 10% FBS Superior, 200 μM L-glutamine, 1% nonessential amino acids, and 100 mM β-mercaptoethanol. All reagents were obtained from Biochrom (Jena, Germany), PAA (Birmingham, UK), and Invitrogen (Thermo Fisher Scientific, Regensburg, Germany). Cells were maintained at 37 °C in a humidified atmosphere containing 5% CO_2_.

### 2.4. Isolation and Characterization of Human Spermatogonial Stem Cells

Spermatogonial cells were separated from the somatic cell monolayer by gentle washing with culture medium. The cell suspension was prepared by careful resuspension and seeded onto 3.6 cm diameter culture plates. Using a Zeiss inverted microscope with a preheated (36 °C) working platform, individual cells were collected at 20× magnification using a micromanipulation pipette.

Spermatogonia were identified by their distinctive morphological characteristics: spherical shape, prominent nucleus-to-cytoplasm ratio, diameter of 7–13 μm, and a characteristic bright cytoplasmic ring between the rounded nucleus and outer cell membrane. The identity of SSCs was confirmed morphologically and by marker expression. Isolated cells were immunostained for SSC markers (GPR125, THY1, and UCHL1) and counterstained for fibroblast-specific proteins (VIM and FSP1). In addition, CD49f^+^ cells were sorted using FACS, and over 85% co-expressed THY1. No fibroblast markers were detected in this sorted population. To confirm germline identity, immunocytochemical analysis was performed using established SSC markers. While DDX4 (VASA) is known to be expressed primarily in spermatocytes and round spermatids rather than in undifferentiated spermatogonia, we used it in conjunction with other markers to assess the germline nature of the isolated cells. This approach allowed us to distinguish spermatogonia from somatic contaminants, with the understanding that DDX4 expression is indicative of more differentiated germ cells. Additional markers specific for SSCs (e.g., [insert relevant markers used, e.g., GPR125, PLZF, or others]) were used to support the identification of spermatogonial stem cells and to reduce fibroblast contamination.

### 2.5. Collection of Single Cells

To examine cellular heterogeneity within colonies, conventional haGSC colonies, human embryonic stem cell (hESC) colonies, and rapidly proliferating human fibroblast (hFib) colonies were enzymatically dissociated into single-cell suspensions. Individual cells were then isolated using manual micromanipulation under a stereomicroscope. This single-cell selection strategy was employed to enable high-resolution analysis of germline- and pluripotency-associated gene expression profiles. The goal was to identify subpopulations within haGSC colonies with favorable transcriptional characteristics and to establish colonies exhibiting optimal expression of key germline and stemness markers.

### 2.6. Immunocytochemistry

Cells were cultured on 24-well plates for 24 h and fixed with 4% paraformaldehyde. After washing with PBS, samples were permeabilized with 0.1% Triton X-100 in PBS for 10 min and blocked with 1% BSA in PBS for 30 min at room temperature. Primary antibodies were applied and incubated overnight at 4 °C. Following 3 washes with PBS (10 min each), cells were incubated with species-specific secondary antibodies conjugated to fluorochromes for 1 h at room temperature. Nuclei were counterstained with 0.2 μg/mL DAPI for 5 min before mounting with Mowiol 4-88 reagent. For negative controls, primary antibodies were omitted. Labeled cells were examined using a confocal Zeiss LSM 700 microscope, and images were captured with a Zeiss LSM-TPMT camera (Zeiss LSM 880, Munich, Germany).

### 2.7. Microarray Analysis

RNA was extracted from hESC line H1 (positive control), long-term haGSC cultures, short-term spermatogonia, and testicular fibroblasts using the RNeasy Mini Kit (Qiagen, Hilden, Germany). RNA was amplified using the MessageAmp aRNA Kit (Ambion, Thermo Fisher Scientific, Germany). Each sample consisted of 200 cells collected via micromanipulation, with cells lysed in 10 μL of RNA direct lysis solution and stored at −80 °C. Samples were analyzed at the University of Tübingen Hospital using Affymetrix Human U133 + 2.0 Genome oligonucleotide arrays. Raw data (CEL files) were processed by MicroDiscovery GmbH (Berlin, Germany) for biostatistical analysis and normalization [1,2,8,10,18,19,20,21]. RNA was extracted from hESC line H1 (positive control), long-term haGSC cultures, short-term spermatogonia, and testicular fibroblasts using the RNeasy Micro Kit (Qiagen, Hilden, Germany) to accommodate low-input samples. Each sample consisted of 200 cells, manually collected using micromanipulation. These cells were lysed in 10 μL of RNA lysis buffer and stored at −80 °C.

To ensure sufficient RNA for microarray hybridization, we applied the MessageAmp™ aRNA Kit (Ambion, Thermo Fisher Scientific), which is specifically optimized for amplifying mRNA from ultra-low input amounts (as low as 10–100 cells). The resulting aRNA underwent quality control assessment using an Agilent Bioanalyzer and Nanodrop spectrophotometer to ensure RNA integrity and quantity were suitable for hybridization. Amplified RNA was then analyzed using Affymetrix Human U133 + 2.0 Genome oligonucleotide arrays. Raw CEL files were processed and normalized by MicroDiscovery GmbH (Berlin, Germany) for downstream bioinformatics.

### 2.8. Collection of Single Cells by Per-Cell Basis 

We used enzymes to separate a conventional haGSC and hESC colony and a rapidly growing hFibs colony into individual cells to investigate the cells within the haGSC colony. Next, we used a micromanipulation technique to pick out specific cells by hand, and we looked at their gene expression levels on a per-cell basis. This approach was developed to gather information on the particular cellular properties of critical genes associated with germline and pluripotency. The aim was to examine the variation in gene expression among selected cells from a representative haGSC colony. Additionally, the goal was to grow colonies with the best germ and pluripotency-related gene expression patterns.

To study heterogeneity in SSC and haGSC cultures, individual cells were isolated using a micromanipulation system mounted on a Zeiss inverted microscope with a 36 °C heated stage. Cells were selected based on well-defined morphological criteria: round cell shape, distinct nuclear boundary, prominent nucleus-to-cytoplasm ratio, and diameter between 7 and 13 μm.

### 2.9. Top Genes Based on the Semantic Similarities of GO Terms

We then used the “GOSemSim” software (Version 2.34.0), which examines the semantic similarity of GO words provided for the genes (also called Friends analysis), to determine the functional similarity between the common differentially expressed genes (DEGs). In the Friends analysis, the top 9 DEGs were deemed hub genes because to their high average functional similarity.

### 2.10. Construction of PPI Network

To investigate the relationship between DEGs, the STRING [22] database (https://www.string-db.org/, accessed on 22 June 2024) constructed the PPI network. After that, we used Cytoscape [23] (v3.7.2) to see the data, then cytoHubba (based on node degree) to find the top 9 DEGs. We used PPI networks and Friends analysis to sort the DEGs into the most important ones, and then we looked for genes involved in spermatogenesis to use as hubs [14,15,16,17,24,25,26,27,28,29]. After constructing the PPI network using STRING, we used cytoHubba (v3.7.2) within Cytoscape to rank hub genes using multiple topological algorithms, including node degree, Maximal Clique Centrality (MCC), Betweenness Centrality, and Edge Percolated Component (EPC). The consensus hub genes were identified based on overlapping results from at least three methods, ensuring more biologically robust selection than node degree alone.

To ensure robust identification of key hub genes in the PPI network, we used cytoHubba in Cytoscape v3.7.2 and applied multiple ranking algorithms: Degree, MCC (Maximal Clique Centrality), and MNC (Maximum Neighborhood Component). Top-ranked genes from each method were compared, and overlapping genes from at least two algorithms were considered consensus hub genes. These were used for functional annotation and further enrichment analysis. The top nine differentially expressed genes (DEGs) were selected based on their high average functional similarity scores in the Friends analysis using GOSemSim. This cutoff was chosen to focus on the most biologically relevant hub genes with strong functional relationships, enabling a more targeted and manageable downstream analysis of key gene interactions involved in spermatogonial stem cell biology and oxidative stress pathways.

### 2.11. WGCNA

Using the R package WGCNA (version 1.73, https://cran.r-project.org/web/packages/WGCNA/index.html, accessed on 18 September 2024) [30], we examined the coexpressed gene module and the hub module that were associated with SSCs. Nine hundred and fifty-eight genes unique to SSCs were chosen for the WGCNA network. The matching dissimilarity was converted from adjacencies between all the filtered genes using a power of β = 9. Cytoscape v3.7.2’s plug-in MCODE was used to display the hub module and genes, with a cut-off MCODE score of 4.5 or above. Following that, the genes in the hub module were annotated and their pathways enriched using DAVID (false-discovery rate, FDR < 0.05). The analyses of the KEGG and Gene Ontology (GO) pathways were then plotted using the R package GOplot v1.0.2 (https://cran.r-project.org/web/packages//GOplot/, accessed on 26 June 2024).

### 2.12. Models Set Up

For model construction, we used 80% of the data as training set and the remaining 20% as testing set. For this purpose, we used eight different ML models for outcome prediction: logistic regression, XGBoost, AdaBoost, decision trees, support vector machines (SVMs), multilayer perceptions (MLPs), and support-vector networks (also known as support-vector networks).

To make the machine learning models more stable, we used Scikit-learn (version 0.23.2) and 5-fold cross-validation. We utilized the following six metrics to measure the model’s efficacy: When the value that is seen (measured) closely matches the value that is really there, we say that the amount is accurate. A narrower margin of error indicates that the measurement is more accurate. The term “precision” describes the proportion of returned instances that are relevant. Recall (sensitivity) is the proportion of important events that were successfully retrieved. The specificity of this test is defined as the proportion of individuals who did not have the disease (according to the “Gold Standard”) but nonetheless had a negative result. The F1 score may be calculated by summing the recall and accuracy harmonically. Analysis of the area under the receiver operating characteristic curve (AUC) allows for the selection of potentially optimal models and the elimination of subpar ones without requiring the description of the cost context or the class distribution. It is easy to see how receiver operating characteristic (ROC) analysis and cost–benefit analysis go hand in hand when deciding how to differentiate.

### 2.13. Analysis of Human Metastatic and Cell–Cell Interactions

Cell type identification markers were sourced. Similarly to the methods detailed for the mouse syngeneic investigations, we used the same pre-processing and categorization techniques. First, we extracted T cells from the whole dataset; next, we classified them into subgroups. The anticipated T cells were then divided into several groups according to their characteristics. The lack of CD4− markers and the expression of CD8A+ and CD8B+ were the defining features of CD8+ positive cells. CD8−, CD8B−, CD4+, FOXP3−, and CD25− cells were used to classify T-helper cells. The regulatory T cells were classified as CD8A−, CD8+, CD4+, FOXP3+, and CD25+ cells. Its method of classification was the same as the one described before. Following the steps indicated earlier, we computed interaction scores and determined their significance.

### 2.14. Development and Evaluation of Differentiation Models

For model construction, 80% of the data were used as the training set, and the remaining 20% served as the testing set. We evaluated eight machine learning algorithms selected based on their widespread use and capability to model complex relationships in biological datasets. These models included logistic regression, XGBoost, AdaBoost, decision trees, support vector machines (SVMs), multilayer perceptrons (MLPs), and support-vector networks.

### 2.15. Statistical Analysis

Data distribution was assessed using the Shapiro–Wilk test. For normally distributed data with equal variances, comparisons between two groups were performed using Student’s *t*-test. For data not meeting these assumptions, the non-parametric Mann–Whitney U test was applied. For multiple group comparisons, Kruskal–Wallis test followed by post hoc Dunn’s test was used as appropriate. Statistical significance was set at *p* < 0.05. All analyses were conducted using [SPSS version 1.3].

## 3. Results

### 3.1. HSSCs Selection

**Figure 1:**
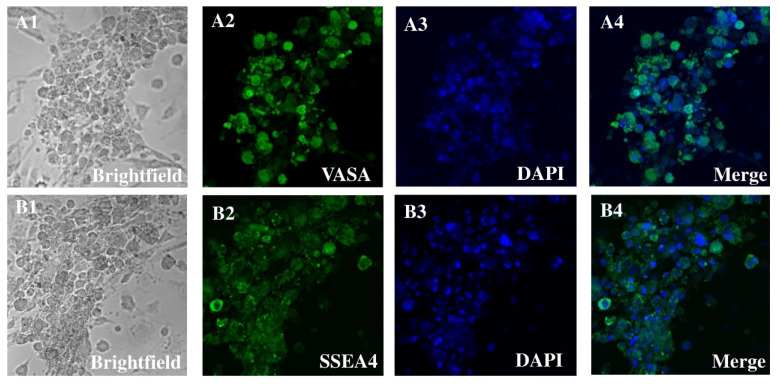
Human spermatogonia cultured in vitro after matrix and CD49f selection. (**A**) The typical morphology of patient 200’s spermatogonia during culture. In every cell culture, there are connected spermatogonia in pairs, chains, small groups, or colonies. (**B**) Inactivated CF1 feeder cells were used to sustain cells grown for a prolonged period of time. Human fibroblasts were absent from the cultures. VASA and SSEA4-positive somatic cells were absent from purified germ cell cultures (Scale bar: 25 μm).

Matrix selection (especially collagen nonbinding/laminin binding) and CD49f-MACS were used to separate and concentrate spermatogonia from orchiectomies performed to collect patient data pertinent to SSC cultures. In the early cultures, positive DDX4 (VASA) and SSEA immunocytochemistry indicated that spermatogonia were most likely the preponderant cells in the chosen cell types. The primary justification for this was the spherical shape, together with its size of around 6–12 μm and the high ratio of nucleus to cytoplasm. A bright cytoplasmic ring between the round nucleus and the outside cell membrane is a telltale sign of this ratio. Connected by intercellular bridges, all cell cultures displayed spermatogonia in pairs, chains, and small clusters. The cultures included many kinds of cells, including larger ones with a diameter of 12–14 μm. The cells were less densely packed with nucleus to cytoplasm and had an oval shape. The htFibs level was much lower in the unselected cell group. The htFibs were effectively isolated from the nonselected cell fractions after they showed significant proliferation in primary cell cultures. Spermatogonial cultures without htFibs are shown in Figure 1.

### 3.2. Gene Expression in SSC Versus Fibroblast

**Figure 2:**
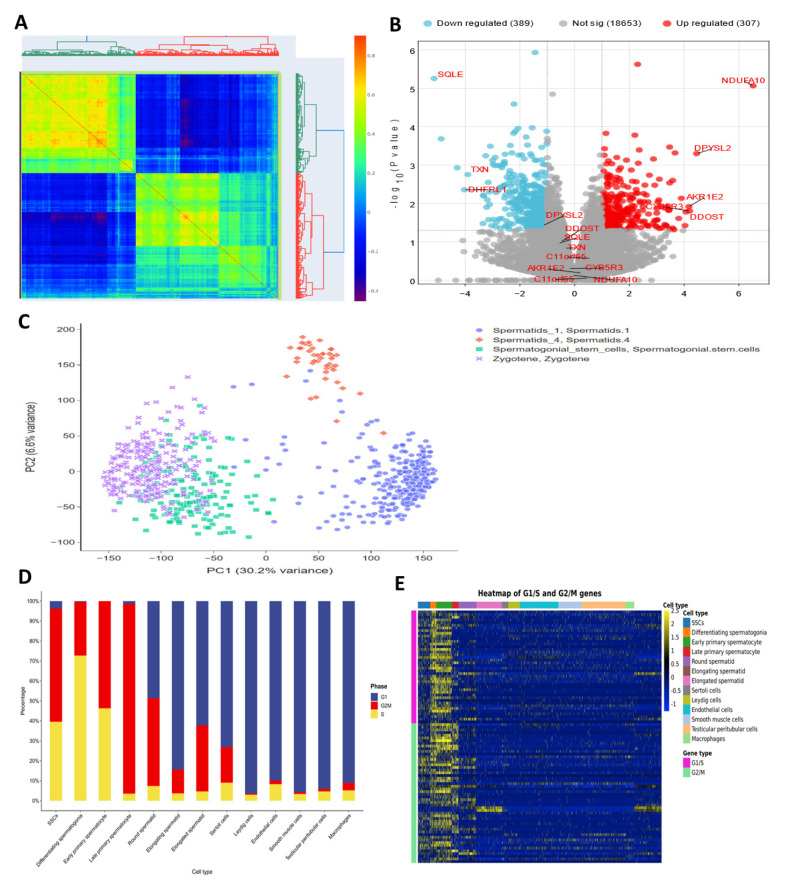
Analysis of gene expression by microarray. (**A**) Correlation plot of SSCs and fibroblasts, (**B**) volcano plot for differentially expressed genes based on microarray analysis, (**C**) PCA analysis in spermatogenesis stages cells, (**D**) Box plot to show the cell cycle stage in spermatogenesis cells, and (**E**) Heat map for showing the cell cycle stage in spermatogenesis cells.

About 526 genes involved in oxidative stress were examined using a microarray. Using microarray analysis, we discovered three types of fibroblasts and three types of SSCs that had elevated genes and three types that had downregulated genes. You may see this data in the figure. Genes DDOST, CYB5R3, NDUFA10, AKR1E2, DPYSL2, and C11orf65 showed overexpression in three SSC human samples analyzed by microarray, while TXN, SQLE, and DHFRL1 showed downregulation (Figure 2).

### 3.3. PPI Network of Oxidative Stress-Related Hub Genes

**Figure 3:**
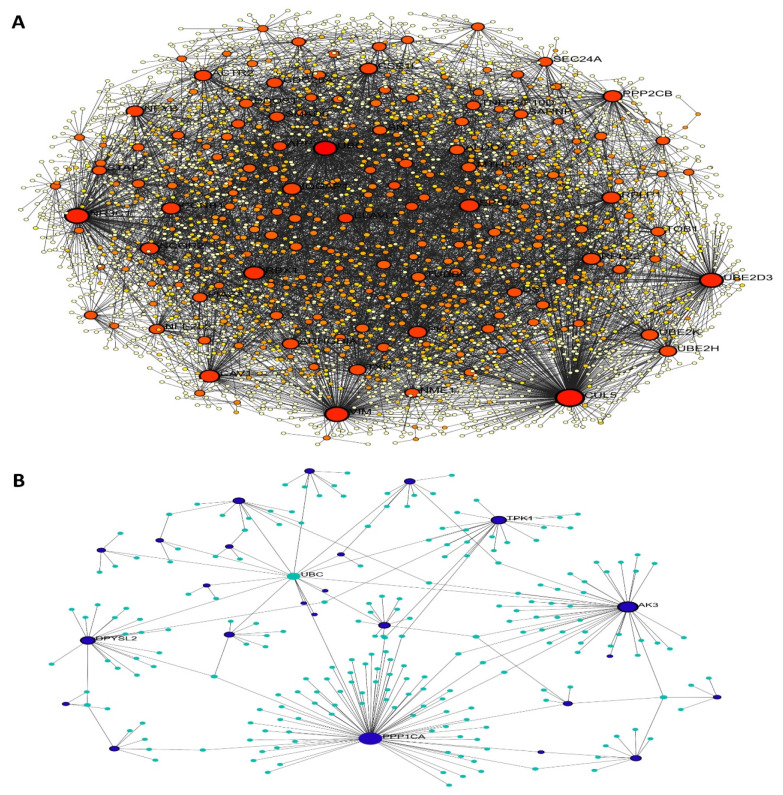
Protein–protein interaction and signaling pathway. (**A**) PPI network of the core genes was established using the STRING online database. (**B**) An investigation of the PPI of the oxidative stress-related hub genes was conducted to ascertain their participation. The red nodes represent genes with a high MCC score, while the yellow nodes indicate genes with a low MCC value.

Using information from the STRING database, a PPI network was built to represent DEGs in SSCs. The results of this study placed 168 genes into the PPI network. With 67 nodes and 210 edges, the PPI network showed a *p*-value for PPI enrichment that was lower than 0.01. Using FunRich, a network was built out of 30 DEGs and the genes that were nearby. The important modules were then located using the MCODE plugin. Modules 1–4 were chosen for functional clustering based on their MCODE scores: 2—(MCODE score = 21.826), 3—(MCODE score = 3), and 4—(MCODE score = 2.8). A KEGG pathway analysis was conducted for every module using DAVID. From the cytoHubba plugin, twenty-one hub genes related to spermatogenesis were identified. These genes include GALNT10, CECR1, B3GALT6, GPX1, CROT, AK3, CA12, HS3ST3A1, PDE6D, AK9, TPK1, MSRA, PCYOX1, GLB1, ECHDC1, MOXD1, PI4KB, and SMPD1. The PPI network of these key genes was constructed using the STRING online database, based on their elevated degree scores. We also used FunRich software (version 3.1.4) to build the core gene and related gene interaction network. The hub genes’ PPI network had nine nodes and thirty-three edges. A high degree of clustering was indicated by the average network local clustering coefficient of 1, which was 1. A significant enrichment of protein interactions was shown by the PPI enrichment *p*-value, which was found to be less than 0.01. Figure 3 shows the findings of a gene co-expression study including the 10 hub genes, which further suggests that these genes probably engage in active interactions.

### 3.4. Functional Enrichment Analysis

**Figure 4:**
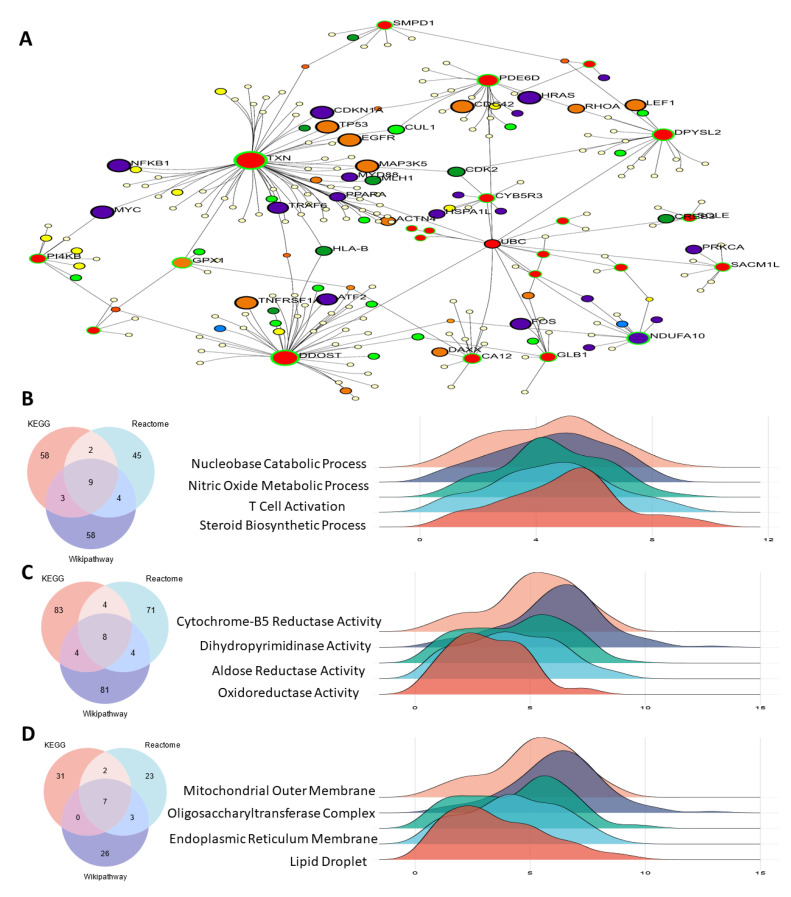
GO analysis of antioxidant gene expression. (**A**) Antioxidant gene expression involved in BP, MF and CC (**B**) biological process category, the five most significant terms from the gene ontology enrichment analysis were associated with steroid biosynthetic process, T cell activation, nitric oxide metabolic process, and nucleobase catabolic process, (**C**) The MF category’s upregulated and downregulated DEGs were linked to oxidoreductase, aldose reductase, dihydropyrimidinase, and cytochrome-B5 reductase activities and (**D**) mitochondrial outer membrane, oligosaccharyltransferase complex, endoplasmic reticulum membrane, and lipid droplet were abundant in the CC category’s upregulated or downregulated differentially expressed genes.

Within the biological process category, the five most significant terms from the gene ontology enrichment analysis were associated with steroid biosynthetic process, T cell activation, nitric oxide metabolic process, and nucleobase catabolic process. These terms denoted the upregulated and downregulated DEGs. The MF category’s upregulated and downregulated DEGs were linked to oxidoreductase, aldose reductase, dihydropyrimidinase, and cytochrome-B5 reductase activities. Gene ontology (GO) terms such as mitochondrial outer membrane, oligosaccharyltransferase complex, endoplasmic reticulum membrane, and lipid droplet were abundant in the CC category’s upregulated or downregulated DEGs (Figure 4).

### 3.5. Weighted Gene Co-Expression Modules

**Figure 5:**
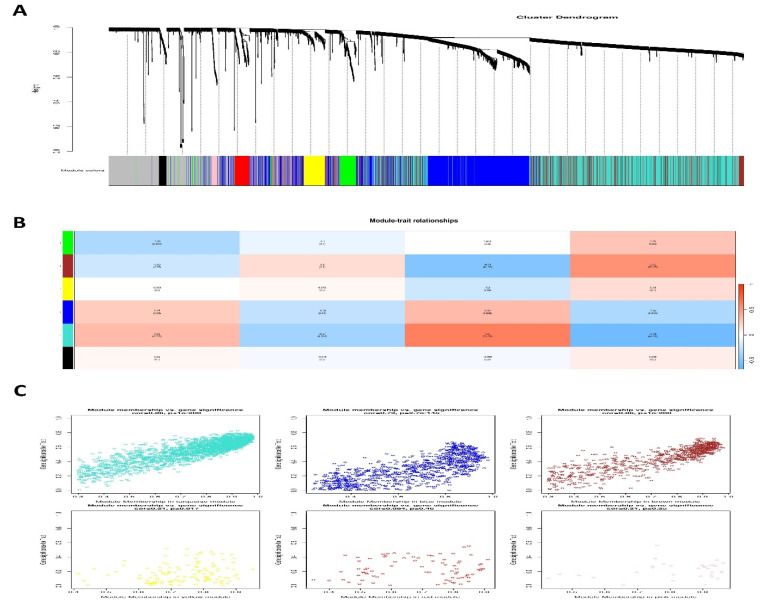
Modules linked to clinical data in SSCs are identified. (**A**) Using hierarchical gene clustering based on 1-TOM, a cluster dendrogram of co-expression network modules is created, giving each module a distinct hue. (**B**) Relationships between module and trait are shown: (**C**) each column denotes a clinical characteristic (normal or cancer), and each row denotes a color-coded module. The corresponding correlation value and related *p*-value are shown in each cell.

To find groups of related genes in SSCs, we used the WGCNA method to build gene co-expression networks. Five separate modules in the SSC, each given its own color, were recently discovered. It must be noted that the color gray was used to indicate a single module that was not included in any cluster. Then, to assess the relationship between modules and characteristics, we made a heatmap. Figure 5 shows the connections between characteristics and modules. According to these studies, the SSC’s brown module establishes the strongest link with healthy tissues (Figure 5 and Supplementary File S1).

### 3.6. Single-Cell RNA Sequencing on Testicular Cells

Using single-cell RNA sequencing and publicly accessible datasets, we characterized the cellular diversity of the testis throughout human development in boys aged 1, 2, and 7 years. Through the identification of oxidative stress-related hub genes during spermatogenesis and the construction of a developmental timetable for testis maturation, we compared various age groups. Prenatal (embryonic weeks 6–16), neonatal (postnatal days 2–7), prepubertal, and peri- to postpubertal ages (11, 13, 14, 17, and 25 years) were all covered by the datasets. For future analysis, a grand total of 82,220 testicular cells were retained.

**Figure 6:**
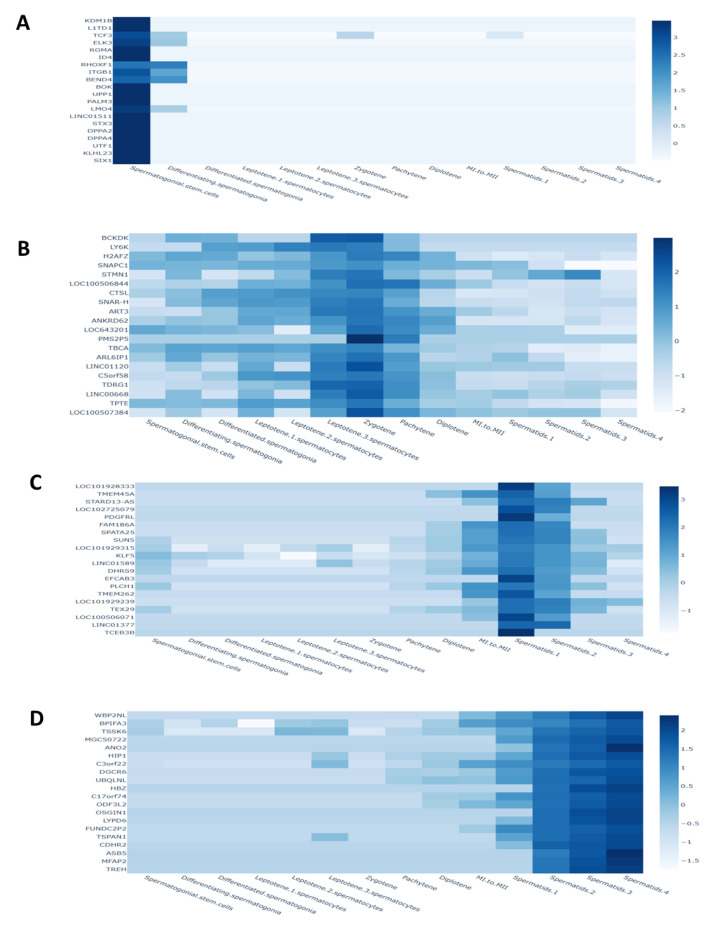
Antioxidant gene expression in SSCs differentiation stages. (**A**) Antioxidant gene expression in SSC, (**B**) Antioxidant gene expression in Leptoten stage, (**C**) Antioxidant gene expression in Spermatid stage 1, and (**D**) in Spermatid stage 4 (mature).

To obtain a better understanding of the variability of germ cells during testis development, 8140 germ cells from clusters 1–4 were re-clustered. Figure 6 shows the results of unbiased clustering using UMAP, which identified fifteen cell clusters that reflect different stages of spermatogenesis. Undifferentiated spermatogonia (Undiff SPG) or SSC/progenitors were found in clusters 1–5, among other markers, by analyzing the expression of UTF1, ID4, and NANOS3. Cluster 6, which represents differentiating spermatogonia, did not express meiotic genes but did express CKIT and STRA8. The spermatocyte-corresponding clusters 7 and 8 showed an overexpression of SYCP3 and SPO11. Clusters 9–15, which included both short and long spermatids (SPtids), expressed TNP1 and PRM2. Figure 6 shows that the latter phases of differentiation, including mature sperm and spermatid, rely on hub genes associated with oxidative stress.

### 3.7. Performance of Antioxidant Gene Expression in SSCs Differentiation Models

**Figure 7:**
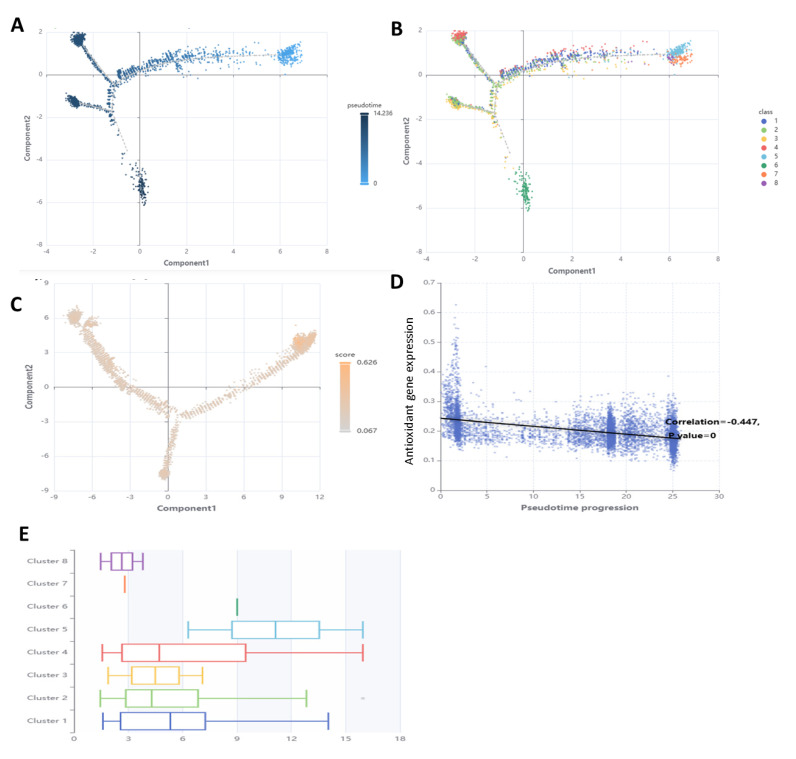
Pseudotime trajectory analysis of spermatogonial cells. (**A**) Pseudotime trajectory analysis reveals two spermatogonial cell developmental trajectories, one representing SSC self-renewal and the other representing SSC differentiation. (**B**) SSC demonstrates cell fate-determining genes associated with spermatogonial cell development. (**C**) Pseudotime trajectory analysis reveals antioxidant gene expression, (**D**) correlation of antioxidant gene expression and pseudotime progression, and (**E**) Faceted plots of pseudotime trajectories at D1, D75, and D150.

In order to forecast the expression of antioxidant genes in SSCs differentiation models, eight ML models were created: support vector machine (SVM), logistic regression, decision tree, random forest, XGBoost, AdaBoost, KNN, and multilayer perceptron. We measured the model’s efficacy using measures including accuracy, precision, recall (sensitivity), specificity, F1 score, and AUC. With an area under the curve (AUC) of 0.741, logistic regression outperformed the other models for SSCs differentiation and antioxidant gene expression (Figure 7 and Supplementary File S2).

### 3.8. Analysis of Single-Cell RNA Sequencing for Antioxidant Gene Expression

**Figure 8:**
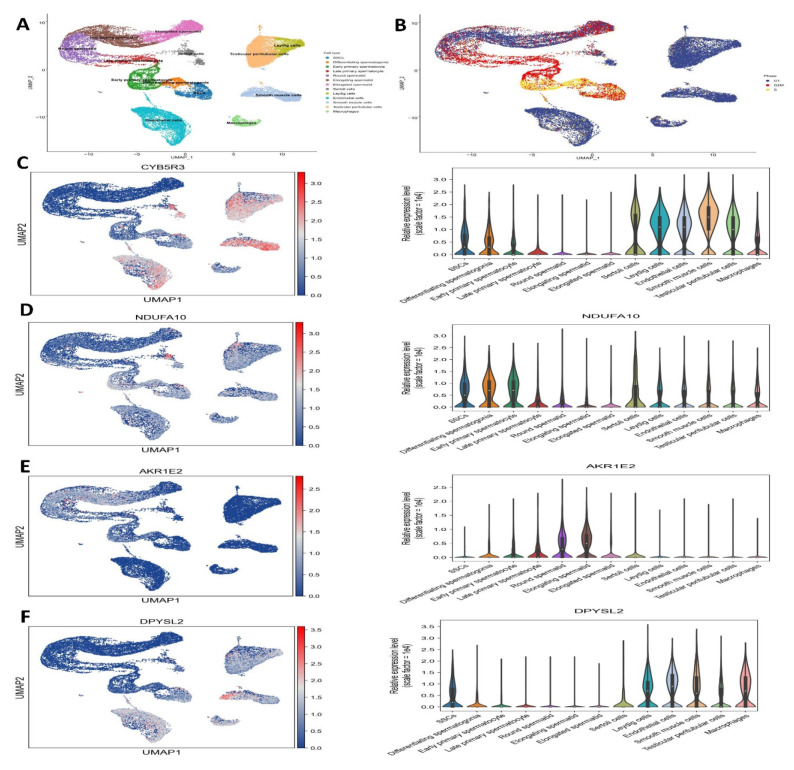
Single-cell RNA sequencing investigation of mouse pup and adult spermatogonia demonstrates similar metabolic patterns to those seen in humans: (**A**) UMAP plot depiction of germ cells from merged single-cell RNA sequencing data, (**B**) cell cluster marker, (**C**) CYB5R3, (**D**) NDUFA10, (**E**) AKR1E2 and (**F**) DPYSL2. These are upregulated, suggesting their potential roles in SSCs.

**Figure 9:**
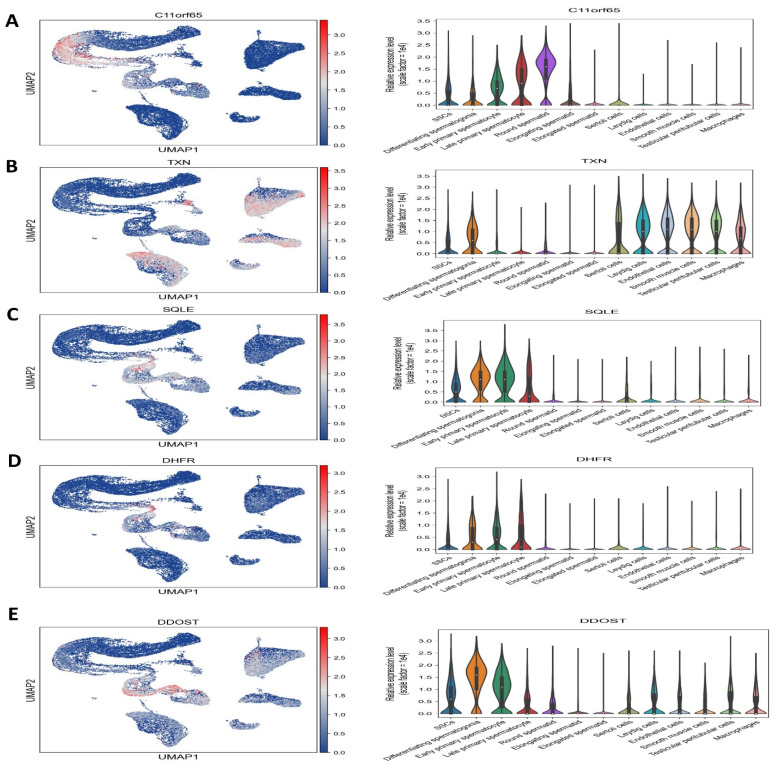
Single-cell RNA sequencing analysis. (**A**) C11orf65, (**B**) TXN, (**C**) SQLE, (**D**) DHFR, and (**E**) DDOST are downregulated, suggesting their potential roles in SSCs.

Through the use of pairwise DEG analysis across age groups, pathway enrichment analysis using GSEA, and consolidation of the six clusters indicating SSC/progenitors into a single cluster, we were able to determine functional modifications during SSC development. A strict false discovery rate cutoff of less than 0.05 was used to find the enriched pathways. Gene expression levels were found to be upregulated in DDOST, CYB5R3, NDUFA10, AKR1E2, DPYSL2, and C11orf65, and downregulated in TXN, SQLE, and DHFRL1, as shown in Figure 8 and Figure 9.

### 3.9. Scoring Cell–Cell Interactions

**Figure 10:**
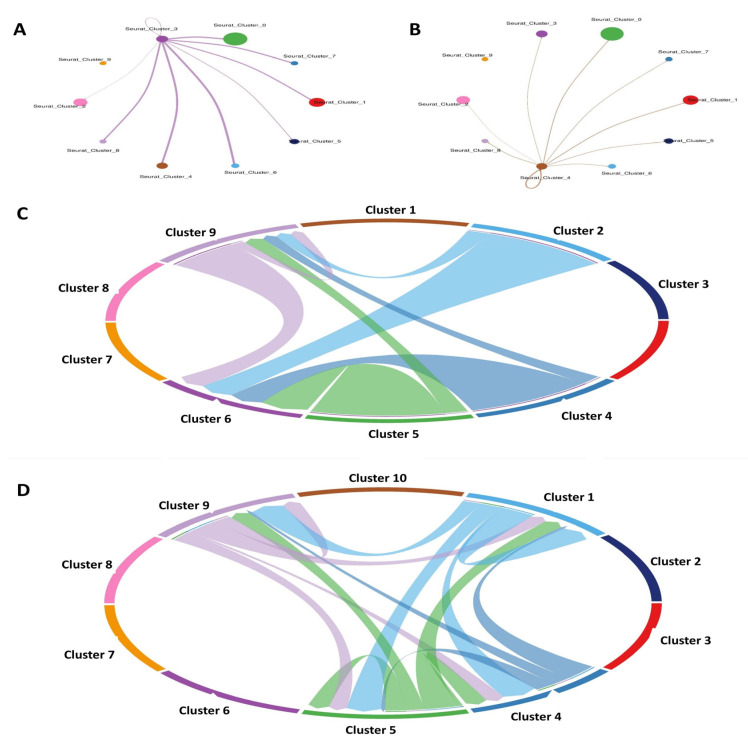
Cell–cell communication analysis. (**A**) network cell–cell communication in early spermatogenesis, (**B**) network cell–cell communication in sperm maturation, (**C**) cell–cell related to early spermatogenesis, (**D**) cell–cell related to sperm maturation.

After different cell types were identified, we looked for possible linkages between all the cells in the tumor microenvironment. Around 1259 interactions that have been verified and supported by science were used. Some examples of the types of interactions that fall under this category are those between integrins in the extracellular matrix (ECM) and various chemokines, cytokines, receptor tyrosine kinases (RTKs), and tumor necrosis factors (TNFs). Given the significance of B7 family members in the various phases of spermatogenesis (Figure 10), we also manually incorporated their identified interactions.

## 4. Discussion

In this study, we successfully enriched and characterized human SSCs from testicular tissue using a combination of CD49f magnetic-activated cell sorting (MACS) and matrix-based selection. This dual strategy significantly improved the purity of SSC cultures by reducing contaminating human testicular fibroblasts (htFibs), a common obstacle in primary testis cultures. Immunocytochemistry confirmed the identity of the selected spermatogonia through DDX4 (VASA) and SSEA expression, while their characteristic morphological features—small spherical shape, high nucleus-to-cytoplasm ratio, and intercellular bridges—further supported their stem cell identity. These findings are consistent with previous reports on human SSC phenotypes and reinforce the robustness of our isolation approach. The gene expression analysis highlighted key differences between SSCs and fibroblasts, particularly in oxidative stress-related genes. Notably, SSCs exhibited upregulation of genes such as CYB5R3, NDUFA10, and DDOST, which are involved in redox homeostasis and mitochondrial function, underlining the unique metabolic demands of SSCs compared to somatic cells. Downregulation of TXN, SQLE, and DHFRL1 further suggests distinct oxidative stress response pathways in SSCs. The PPI network analysis, which identified key hub genes enriched in protein–protein interactions, reveals a tightly interconnected oxidative stress response network crucial for SSC maintenance and spermatogenic progression.

Further functional clustering using MCODE and pathway enrichment analyses via DAVID and FunRich underscored the significance of these DEGs in pathways relevant to SSC biology. For instance, KEGG pathway analysis highlighted roles in steroid biosynthesis [31], nitric oxide metabolism [32], and nucleobase catabolism—pathways vital for germ cell differentiation and testicular microenvironment interactions [33]. Gene Ontology analysis also pointed to mitochondrial and endoplasmic reticulum components as critical subcellular localizations, underscoring the organelle-level complexity of SSC metabolic regulation. The small number of donors (*n* = 3) and their wide age range (23–67 years) present a limitation, as both age and fertility status may influence SSC function and oxidative stress response. Future studies with larger, age-stratified cohorts are required to validate the observed transcriptomic and functional signatures.

The integration of WGCNA and clinical trait association revealed a strong correlation between the brown gene module and healthy tissue, suggesting this module might contain genes critical for maintaining normal SSC function. This analysis provides a systems-level view of the co-regulated gene sets involved in spermatogenesis and presents potential targets for understanding or manipulating SSC behavior. Our scRNA-seq analysis revealed the developmental trajectory and cellular heterogeneity of human germ cells from infancy to adulthood. Importantly, oxidative stress-related genes showed stage-specific expression, with higher activity during later stages such as spermatid maturation [34,35]. This indicates a possible increased demand for redox regulation as cells transition through meiosis and into the final phases of spermatogenesis [36]. The co-expression of antioxidant genes in advanced germ cell stages aligns with prior evidence that oxidative stress management is essential during sperm maturation to prevent DNA damage and preserve fertility [37,38]. A key limitation of this study is the small sample size and broad age range of the donors, which may introduce biological variability and limit the generalizability of the findings. Future studies should involve larger, age-matched cohorts to validate oxidative stress-related transcriptional signatures in SSCs.

These findings align with recent research emphasizing the importance of oxidative stress regulation in SSC biology. For example, studies by Abdulle et al. [39] demonstrated that mitochondrial function and redox homeostasis are critical for SSC self-renewal and differentiation, supporting our observations of upregulated mitochondrial genes such as CYB5R3 and NDUFA10. Moreover, single-cell transcriptomic analyses by Divvela et al. [40] revealed stage-specific antioxidant gene expression patterns during spermatogenesis, consistent with our pseudotime trajectory results showing increased oxidative stress gene activity in late germ cell stages.

Machine learning models, particularly logistic regression, effectively predicted antioxidant gene expression during SSC differentiation, with an AUC of 0.741. This demonstrates the potential utility of computational tools in modeling SSC biology and identifying biomarkers for stem cell potency and differentiation potential. Pseudotime trajectory analysis further clarified bifurcated SSC fates—self-renewal versus differentiation—and the dynamic expression of antioxidant genes along this continuum. This supports the idea that oxidative stress pathways are not merely background metabolic processes but active regulators of fate decisions in SSCs.

Finally, cell–cell communication analyses demonstrated significant interactions between germ cells and their surrounding microenvironment. These included cytokines, integrins, and ECM components that likely influence SSC fate and maintenance. The inclusion of B7 family immune-modulatory molecules also suggests that immune privilege and local immune signaling are critical in germ cell development, an area requiring further exploration [41,42].

Protein oxidation is a critical mechanism through which oxidative stress compromises sperm function and male fertility. Reactive oxygen species (ROS) target amino acids such as cysteine, methionine, and tyrosine, leading to carbonyl group formation and disulfide bonding that disrupts protein structure and function. In spermatozoa, key proteins affected include cytoskeletal elements like actin and tubulin, whose oxidation impairs flagellar integrity and motility—essential for successful fertilization [43,44,45]. Additionally, metabolic enzymes such as creatine kinase and adenylate kinase, which are vital for ATP production, are also vulnerable, resulting in diminished energy availability and reduced sperm function.

Oxidative modifications further disrupt the acrosome reaction by impairing proteins like acrosin and hyaluronidase, which are essential for zona pellucida penetration [46,47,48]. This cascade of oxidative damage translates to impaired fertilization capacity. Proteomic analyses have substantiated these effects by identifying oxidized sperm proteins and associated functional deficits in infertile men [49,50]. Clinical studies support antioxidant therapies as a promising intervention, improving motility and reducing oxidative damage. These insights reinforce our findings, which highlight the central role of oxidative stress pathways—particularly those regulating redox homeostasis and protein integrity—in SSC maintenance and spermatogenic progression [51,52]. Integrating molecular analyses and functional assessments of protein oxidation may further clarify therapeutic strategies for oxidative stress–induced male infertility.

## 5. Conclusions

This study successfully enriched and characterized human spermatogonial stem cells, revealing distinct oxidative stress-related gene expression profiles compared to somatic cells. Key hub genes involved in redox homeostasis and mitochondrial function were identified, underscoring the metabolic uniqueness of SSCs. Our integrative analyses suggest that oxidative stress pathways actively regulate SSC maintenance and differentiation, highlighting potential molecular targets for improving male fertility treatments. Future research with larger, age-matched cohorts is needed to validate these findings and further explore therapeutic strategies targeting oxidative stress in SSCs.

## Data Availability

The original contributions presented in this research are included in the article; further inquiries can be directed to the corresponding author.

## Supplementary Materials

The following supporting information can be downloaded at: https://www.mdpi.com/article/10.3390/antiox14080936/s1, File S1: SSCs Differentiation Models; File S2: WGCNA analysis.
